# Incremental Utility of Awareness of Deficit as a Predictor of Dementia Outcomes

**DOI:** 10.1093/geroni/igaf122.2867

**Published:** 2025-12-31

**Authors:** Brenda Owe, Anthony Robinson, Destiny Weaver, Lisa Rapport

**Affiliations:** Wayne State University, Detroit, Michigan, United States; Wayne State University, Detroit, Michigan, United States; Wayne State University, Detroit, Michigan, United States; Wayne State University, Detroit, Michigan, United States

## Abstract

Subjective report of cognitive function is often used as a proxy for actual cognitive status; however, individuals with cognitive decline frequently underestimate their impairments. Impaired self-awareness of deficits (anosognosia) is important to understand objective function and risk for multiple adverse outcomes. This study examined the incremental contribution of assessing impaired awareness of deficits (anosognosia) over self-ratings of cognitive capacity. Data were obtained from 793 participants (ages 70-110 years) in the Aging, Demographics, and Memory Study supplement to the Health and Retirement Study. Main outcome measures included clinician-report and informant-report dementia rating scales, and global cognitive status assessed using the Mini Mental State Examination (MMSE). Awareness of deficit was operationalized as the difference between self-reported memory and objective performance on a verbal memory test. Self-reported memory showed weak correlation with clinician-report and informant-report dementia rating scales and the MMSE, whereas anosognosia showed medium-to-large correlations with those outcomes. Importantly, anosognosia contributed unique variance in predicting all three outcomes, beyond that accounted for by age, education, gender, and a different objective measure of verbal memory. In contrast, parallel models testing self-reported memory indicated that it was not a significant predictor of dementia rating scales or the MMSE. Both self-perception of cognition and objective cognitive performance are necessary to understand an individual’s awareness of their deficits. Information about self-perception of cognitive status is important to understand subjective well-being; however, the discrepancy between self-perception and objective capacity provided unique information about functional cognitive status, whereas self-report alone did not.

